# Impact of Different Proportions of Purple Rice and Chanterelles Powder on Physicochemical, Sensory, and Starch Digestibility Properties in Wheat Bread

**DOI:** 10.3390/foods14081343

**Published:** 2025-04-14

**Authors:** Yingrui Hu, Yiqing Jia, Zhilin Li, Zhishuang Wang, Ting Wei, Feifei Bi, Yurou Wang, Yuyue Qin, Afsar Khan, Yaping Liu, Guiguang Cheng

**Affiliations:** 1 Faculty of Food Science and Engineering, Kunming University of Science and Technology, Kunming 650500, China; yingruistar@163.com (Y.H.); jiaq7207@163.com (Y.J.); 15308744719@163.com (Z.L.); 15887696256@163.com (Z.W.); wt15912513013@163.com (T.W.); 15288235956@163.com (F.B.); wyr111519@163.com (Y.W.); rabbqy@163.com (Y.Q.); 2 Department of Chemistry, Abbottabad Campus, COMSATS University Islamabad, Abbottabad 22060, Pakistan; afsarhej@gmail.com

**Keywords:** bread recipe, functional characteristics, glycemic index, in vitro digestion, antioxidant capacity, volatile compounds

## Abstract

The breads were produced using the following formulations: (1) purple rice (*Oryza sativa* L.) powder alone at 5% and 10% (*w*/*w*), (2) chanterelle mushrooms (*Cantharellus yunnanensis Chiu*) powder alone at 1% and 2% (*w*/*w*), and (3) four blended combinations of both purple rice powder (5%, 10%) and chanterelle powder (1%, 2%) at varying ratios. Physicochemical, starch digestibility, antioxidant capacity, odor characteristics, and sensory properties were investigated, which are helpful to search for both rich-nutritious and highly acceptable daily food options. Compared to the control bread, the resistant starch content, phenolic content, flavonoid content, and antioxidant capacity were significantly increased, and the hydrolysis index and glycemic index were significantly decreased in experimental breads. Significant differences were found in color, specific volume, texture characteristics, and aroma components in experimental breads. All the experimental breads showed high overall acceptability, and the results indicated that purple rice and chanterelle mushroom powder could be used as high-value ingredients to improve the nutritional profile and reduce the glycemic index of bread. The purple rice and chanterelle mushrooms are natural food ingredients and show new potential to improve the functional properties of breads.

## 1. Introduction

The hidden hunger is a phenomenon in which people tend to intake foods with high energy but lack multiple micronutrients in their daily diet [1]. According to statistics, around 2 billion people are in a condition of hidden hunger around the world, which causes approximately USD 3.5 trillion annual loss [2]. However, many chronic diseases such as diabetes, cardiovascular diseases, and obesity are associated with nutrient deficiency in foods [3]. Dietary interventions to improve the nutrient density in daily diets are one of the most effective strategies for solving the problem of hidden hunger [1]. A study demonstrated that consuming pigmented rice could significantly increase antioxidant capacity to alleviate oxidative stress and inflammation caused by obesity and reduce the risk of obesity caused by lifestyle factors [4]. The consumption of vegetables, mushrooms, whole grains, legumes, and fruits may decrease the risk of diabetes and obesity. Dietary fiber could contribute to appetite suppression by increasing satiety, while both polysaccharides and polyphenols could combine with dietary fat to reduce blood lipid and cholesterol levels [2,5].

Glycemic index (GI) is an indicator used to measure the effect of carbohydrate containing foods on blood glucose concentration. It reflects the speed and magnitude of blood sugar increase caused by foods containing carbohydrates after digestion and absorption [6]. GI value is an important tool for evaluating the sugar raising potential of food, and it serves as a key indicator to evaluate food health in modern nutrition [7]. Foods with low GI value not only slow down the rate of digestion and absorption, thereby reducing postprandial blood sugar fluctuations, but also prolong satiety and control calory intake, ultimately reducing fat synthesis and accumulation [8]. Many low-GI foods are usually rich in dietary fiber and resistant starch, which promotes intestinal motility to prevent constipation and intestine-related diseases [6]. Low-GI ingredients such as beans, whole grains, mushrooms, and vegetables are nutrient dense, which provides comprehensive nutrition to the body and forms a healthy and sustainable eating habits [7].

Purple rice (*Oryza sativa* L.), belonging to the gramineous Oryza plant and a kind of pigmented glutinous rice, is usually cultivated in Asian regions such as China, Japan, and Thailand [9]. Purple rice is a popular food due to its soft texture and rich aroma. It is widely used in daily meals, such as rice dishes and porridge, and is also used to brew liquor [9]. Purple rice is an excellent source of rich and comprehensive nutrients, containing proteins, polyphenols, flavonoids, dietary fiber, and minerals. Specifically, it contains higher levels of anthocyanins, amino acids, and vitamin B than those of ordinary rice [10]. Furthermore, purple rice has a variety of medicinal benefits, such as protecting liver and stomach, invigorating kidney, improving vision, and promoting healthy blood [11]. A study reported that the intake of cooked purple rice could significantly reduce inflammatory factors, improve antioxidant capacity, and lower the risk of lifestyle diseases [12].

The chanterelle mushroom (*Cantharellus cibarius*) is a sub-body of Agaricales which are the most popular consumed mushrooms in Europe, and it is commonly distributed throughout Asia and North America, including Yunnan province in China [13,14]. Chanterelle mushrooms have rich nutritional values such as protein, dietary fiber, vitamins, and minerals, especially vitamin A and D [14,15]. The chanterelle mushroom is a daily food in Europe, which has not only a unique almond aroma but also high medicinal values such as moisturizing the lungs, protecting eyes, and preventing dry skin [16]. Many studies have reported that the chanterelle mushrooms contain many active ingredients like polysaccharide, amino acid, and phenolic acid, which have antioxidant, hypoglycemic, anti-inflammatory, antiviral, and potential anti-cancer effects [16,17]. Mushrooms have always been a popular ingredient due to their special aroma and rich nutrients [18]. Cirlincione et al. (2022) increased the vitamin and polyphenol content of bread by replacing some wheat flour with *Pleurotus eryngii* powder [19]. Similarly, Lu et al. (2018) developed a pasta which could decrease the glycemic index and improve the antioxidant capacity with the addition of shiitake mushroom and porcini mushroom [20].

Bread is a popular daily staple food throughout the world [21]. According to statistics, the production of bread exceeds 9 million tons annually and is increasing year by year [22]. Changes in modern lifestyle and consumer concepts have led to functional bread, developing a hot choice [23]. Compared with white bread, functional breads are rich in nutrients, in addition to high starch content, and also contain more protein, dietary fiber, vitamins, and minerals, which complies with the dietary guidelines for consumers and has become a popular breakfast choice [24]. Pejcz et al. (2024) discovered that fermenting rye dough with *Lactobacillus casei* and *Lactobacillus plantarum* could enhance the antioxidant activity of bread [25]. Moreover, incorporating *Moringa oleifera* leaf powder into wheat flour not only imparts a fruity aroma but also increases the content of protein and minerals in the bread [26]. A bread with the addition of dietary fiber of red potato could reduce glycemic index and increase sensory acceptance was produced, which could prevent metabolic syndrome and type-2 diabetes mellitus [23].

This study aimed to develop functional bread formulations through the incorporation of purple rice and chanterelle mushroom powders. This functional bread could enhance nutritional density through bioactive compounds, modulate glycemic response by altering starch digestibility capacity, and increase antioxidant capacity for chronic disease prevention. By addressing both micronutrient deficiency (hidden hunger) and diet-related chronic conditions through staple food modification, this study demonstrates the feasibility of creating nutritionally bakery products while maintaining consumer acceptability. A comprehensive evaluation was measured in this study, including analyses of chemical composition, physical properties, in vitro starch digestibility, total phenolic and total flavonoids content, antioxidant capacity, volatile gas composition, and consumer acceptance.

## 2. Materials and Methods

### 2.1. Preparation of the Purple Rice and Chanterelle Mushroom Powder

Purple rice (*Oryza sativa* L.) was obtained from traditional terrace-cultivated fields during the 2024 harvest season and purchased from a local farmer, in Yuanyang village, Yunnan, China. The purple rice was crushed into powder with an ultra-fine crusher (DLF-20, Dingli Instrument Co., Ltd., Wenzhou, China) until it passed through a 200-mesh sifter. It was stored at −20 °C until use.

Wild chanterelle mushrooms (*Cantharellus yunnanensis Chiu*) were harvested during the 2023 rainy season (July–August) and purchased from the mushroom trading market in Kunming, Yunnan, China. The fresh mushrooms were cleaned and freeze-dried in a vacuum freeze dryer (XU-18N-80C, Xiui-LAB Co., Ltd., Shanghai, China). The dried mushrooms were milled into powder and passed through a 200-mesh sieve. It was stored at −20 °C until use.

### 2.2. Preparation of Bread

The bread was prepared according to the secondary fermentation method as detailed in reference [27]. The bread preparation incorporated purple rice and chanterelle mushrooms powder in different proportions instead of wheat flour, as shown in Table 1. All ingredients were added to the Kitchen mixer (M6, Hauswirt Baking Electrical Appliance Co., Ltd., Qingdao, China) and kneaded for 20 min until smooth spherical dough was formed. The dough was fermented for two hours in a fermentation incubator (YH-12, WANMAI Machinery Co., Ltd., Foshan, China) at 22 °C and 30% relative humidity. Then, the fermented dough was divided into two equal parts and kneaded into smooth balls. The balls were fermented for 50 min at 30 °C and 70% relative humidity. Finally, the bread was baked for 20 min at 180 °C in an oven (MDC-JX-CP20, Maidachu Energy-Saving Technology Co., Ltd., Dongguan, China). The bread was placed in a self-sealing bag after the breads cooled completely.

### 2.3. Bread Quality

#### 2.3.1. Determination of Bread Composition

The moisture and ash content in bread were determined by the AOAC official method 925.09 [28]. The total protein and total fat of bread were determined using AOAC official methods 978.04 [29] and 920.85 [30], respectively. The contents of total dietary fiber (TDF), soluble dietary fiber (SDF), and insoluble dietary fiber (IDF) were quantified using the AOAC official method 985.29 [31]. Moreover, the content of total carbohydrates was determined by the difference method, while the calorie was calculated by Atwater factor according to the following formulas [32]:

$$Total\;carbohydrates\left(\%\right)=100-({Moisture}_{\%}+{Ash}_{\%}+{Protein}_{\%}+{Fat}_{\%}+{Fiber}_{\%})$$

$$Energy\;\left(kcal\right)=4*\left(g_{protein}+g_{carbohydrates}\right)+9*g_{fat}+2*g_{fiber}$$

#### 2.3.2. Specific Volume

The specific volume of bread was calculated according to the rape seed displacement method (AACC 10-05.01) [33]. The specific volume was calculated by the following formula:

$$W=\frac{V}{M}$$

where *W* is the specific volume of bread (mL/g), V is the volume of rape seed (mL), M is the weight of bread (g).

#### 2.3.3. Colorimetric Analysis

The colors of core and crust of each bread were measured using color measurement with Chroma Meter (CR-400, Konica Minolta lnc., Tokyo, Japan), and the values of L (lightness), a (redness), and b (yellowness) were obtained. Then, the color differential index (∆*E*) was calculated through the following formula [33]:

$$∆E=\sqrt{{\left(L^{*}-L\right)}^{2}+{\left(a^{*}-a\right)}^{2}+{\left(b^{*}-b\right)}^{2}}$$

where $L^{*}\left(91.29\right);\;a^{*}\left(-0.73\right);\;b^{*}(3.76)$ represent the measurement values of the standard white plate.

#### 2.3.4. Texture Analysis

The bread was cooled at room temperature for 2 h and was cut into slices 1.5 cm thick. A slice of bread was placed on the platform of the texture analyzer (TA-XT Plus, Hengpin Technology Co., Ltd., Shanghai, China) and a P/36R probe was used for testing. The speed of P/36 R probe was 1.0 mm/s before the test and 5.0 mm/s after the test, the compression degree was 60%, and the induction force was 6 g [34]. The hardness, springiness, chewiness, and cohesiveness of the bread were measured, and each sample was tested 3 times.

### 2.4. Total Phenolic and Total Flavonoid Contents

The total phenolic content (TPC) and total flavonoid content (TFC) were determined according to Chen et al. [35], with slight modifications. A 5 g dry sample of bread was immersed in 80% methanol. The sample was ultrasonically treated for 30 min 3 times. The supernatant was taken out by centrifugation for 15 min at 4000 r/min.

The total phenolic content was determined using the Folin–Ciocalteu method, and the results were expressed as mg gallic acid equivalent (GAE)/g dry bread. In short, the sample extract was added in Folin phenol reagent for 1 min at room temperature, then added to Na_2_CO_3_ (20%, *m*/*v*) solution reacted for 10 min with 70 °C. The solution was taken out and the absorbance was measured at 765 nm.

The result of flavonoids content was expressed as mg rutin equivalent (RE)/g dry bread. In brief, sample extract, ethanol (60%, *m*/*v*), and NaNO_2_ (5%, *m*/*v*) were added in test tube and kept for 8 min at room temperature. Then, Al(NO_3_)_3_ (10%, *m*/*v*), NaOH (10%, *m*/*v*), and ethanol (60%, *m*/*v*) were added in, and absorbance was measured at 510 nm after reacting for 8 min at room temperature.

### 2.5. Determination of Antioxidant Capacity

The method of ABTS and DPPH radical scavenging capacity was determined according to Chen et al. [35] with slight modifications. A total of 5 mL ABTS solution (7 mM) and 88 μL potassium persulfate solution (40 mM) was mixed and stored for 16 h in the dark at 25 °C, then diluted 100 times with anhydrous ethanol to form ABTS working fluid. Then, a 30 μL sample solution and 240 μL ABTS working fluid were added in a 96-well plate and reacted in the dark for 10 min at 30 °C. Then, absorbance was measured at 734 nm.

A total of 39.4 mg DPPH was added in 100 mL methanol and diluted 10 times with methanol to form a DPPH working fluid (0.1 mM). Then, a 0.5 mL sample solution was mixed with 2 mL DPPH working fluid and shaken continuously for 30 min at room temperature. Then, absorbance was measured at 517 nm.

### 2.6. In Vitro Starch Digestion Analysis

The in vitro starch digestion analysis was determined using the method described by Liu et al. [33] with slight modifications. Firstly, oral digestion was simulated with weighed 100 mg of freeze-dried bread powder, adding 3 mL distilled water and 2 mL amylase balanced with Na_2_CO_3_ (pH = 7, 40 U/mg). The mixture was put into a constant temperature shaker (THZ-82A, Xuri Instrument Factory, Changzhou, China) for 5 min at 37 °C with 150 r/min. Then, gastric digestion was simulated, adding 10 mL gastric protease (2500 U/mg, pH = 1.5) balanced with 0.02 M HCl and shaking it for 30 min at 37 °C with 150 r/min. Finally, small intestine digestion was simulated, adding 3 mL of trypsin (1000 U/mg, 1 mg/mL) in EDTA. When the samples were digested at 0, 20, 60, 90, 120, and 180 min, 1 mL of the sample solution was taken and boiled for 10 min to prevent digestion. After cooling down, the glucose content was tested using a glucose reagent kit (THZ-82A, Xuri Experimental Instrument Factory, Changzhou, China).

The determination of glucose content, digestible starch content, and starch hydrolysis rate was calculated according to the following formulas [36]:

$$Glucose\;content(mg/dL)=\frac{A_{Sample}}{A_{standard}}\times 0.55\times 18$$

Digested starch in the sample(mg/dL)=glucose content×0.9

$$Starch\;hydrolysis\;rate(\%)=\frac{digested\;starch\;in\;the\;sample}{Total\;starch\;of\;the\;sample}\times 100$$

According to the in vitro simulated digestion kinetics model of Liu et al. [31], a curve was created with hydrolysis time and starch hydrolysis rate. The integral area under the curve (AUC) was used to calculate the expected glycemic index (eGI) and hydrolysis index (HI) of the bread.

$$HI=\frac{AUC\;of\;the\;bread}{AUC\;of\;the\;control\;bread}\times 100$$

eGI=9.198+0.862×HI

### 2.7. Calculation of Rapidly Digestible, Slowly Digestible, and Resistant Starch

Based on the enzymatic hydrolysis time, starch could be divided into three types: rapidly digestible starch (RDS), slowly digestible starch (SDS), and resistant starch (RS). According to the method described by Liu et al. [36], the starch content with different characteristics could be calculated. The calculations were performed using the following formulas:

$$RDS(\%)=\frac{(G_{20}-G_{0})\times 0.9}{TS}\times 100$$

$$SDS(\%)=\frac{(G_{120}-G_{20})\times 0.9}{TS}\times 100$$

$$RS\left(\%\right)=\frac{\left(TG-G_{0}\right)\times 0.9}{TS}\times 100-(RDS+SDS)$$

where G_0_ is free glucose content at 0 min, G_20_ and G_120_ are the contents of glucose hydrolyzed after 20 and 120 min, respectively. TG (mg) is the total glucose content in the sample, TS (mg) is the total starch content in the sample, and 0.9 is a conversion factor that converts glucose content into starch content.

### 2.8. Determination of Volatile Organic Compounds in Bread

The volatile organic compounds in the bread were characterized by using a gas chromatography–mass spectrometer (GCMS-QP2010, SHIMADZU, Kyoto, Japan), using the method described by Pellacani et al. [37]. The 5 g bread samples were homogenized and put in a 20 mL sealed sampling bottle and then extracted by using solid-phase microextraction (SPME) fiber assembly for 10 min at 60 °C. Furthermore, SPME fiber assembly polydimethylsiloxane (PDMS, Merck, Kenilworth, NJ, USA) was inserted into the sampling bottle for 30 min at 60 °C from the headspace, and volatile component analysis was performed using GC-MS.

The PDMS fiber was transferred to the split–splitless inlet and desorbed for 2 min at 250 °C. A DB-5MS capillary column (30 m × 0.25 mm × 0.25 μm) was used, and the carrier gas was helium with a constant flow rate of 1 mL/min. The temperature of the gas chromatography oven was programmed at 50 °C for 5 min, ramped 5 °C/min to 200 °C, then ramped 20 °C/min to 250 °C and held for 10 min. The detection of a mass spectrometer was performed with electron impact ionization at 70 eV, operated with a range of 50–450 *m*/*z*, and the temperature of the transfer line was 280 °C.

The volatile organic compounds in bread were identified and compared with databases NIST05 and NIST21, and the compounds with more than 85% match were retained. The relative content of each compound was calculated by the percentage of the total peak area and the results expressed as three replicates.

### 2.9. Sensory Evaluation

After cooling, the baked bread was cut into slices, and then 5 central portions of bread were randomly selected. A quantitative descriptive analysis (QDA) was conducted to assess the shape, color, fluffiness, texture, and taste [38]. The sensory profiling was followed by ISO 13299:2016 [39] and China National Standard GB/T 12311–2012 for sensory analysis methodology [33]. The evaluation was conducted in a controlled food sensory laboratory by a trained panel of 30 assessors (including 15 males and 15 females, aged 18–50) from the Faculty of Food Science and Engineering, Kunming University of Science and Technology. All the panelists had prior sensory evaluation experience and received a 20 h standardized assessor training program, which contained basic taste recognition, texture assessment, and protocol familiarization. To minimize carryover effects, panelists rinsed their mouths with purified water and observed for 1 min between sample evaluations.

### 2.10. Statistical Analysis

The experimental values of chemical composition, SV, HI, eGI, RDS, SDS, RS, specific volume, color, and textural characteristics were expressed as mean ± standard deviations and analyzed by SPSS 27.0 software; significance was analyzed with one-way ANOVA and Duncan’s multiple range tests (*p* < 0.05). The standard deviations of total phenolic and flavonoid content, DPPH and ABTS, starch hydrolysis rate, and sensory evaluation value were expressed as error bars, and significance was analyzed with one-way ANOVA and Duncan’s multiple range tests (*p* < 0.05). The volatile organic compounds data were calculated by the percent of the total peak area and analyzed with one-way ANOVA.

## 3. Results and Discussion

### 3.1. Nutritional Analysis

**Figure 1:**
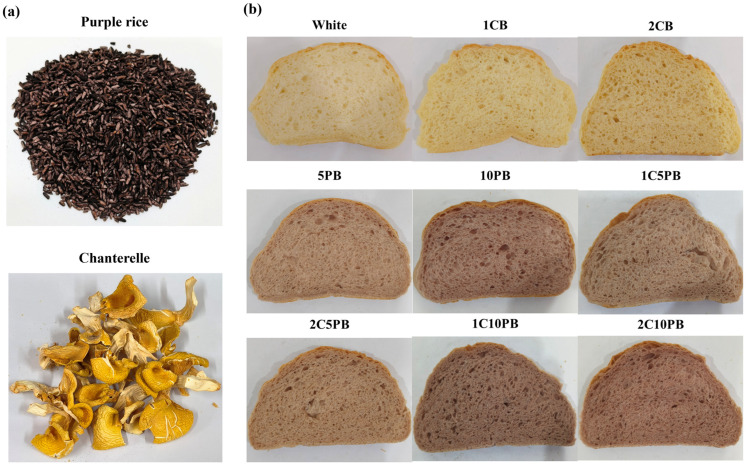
The material of purple rice and chanterelle mushrooms (**a**); the slices of bread with different proportions of chanterelle and purple rice powder (**b**). 1CB: bread with 1% chanterelle mushroom powder; 2CB: bread with 2% chanterelle mushroom powder; 5PB: bread with 5% purple rice powder; 10PB: bread with 10% purple rice powder; 1C5PB: bread with 1% chanterelle mushroom and 5% purple rice powder; 2C5PB: bread with 2% chanterelle mushroom and 5% purple rice powder; 1C10PB: bread with 1% chanterelle mushroom and 10% purple rice powder; 2C10PB: bread with 2% chanterelle mushroom and 10% purple rice powder.

According to the recipe in Table 1, nine kinds of bread are shown in Figure 1, which include different proportions of purple rice and chanterelle powder instead of wheat flour. The erythritol was selected as the sweetening agent in this study due to its notable functional benefits, including zero glycemic index and low caloric content. These properties enable effective sweetness delivery for maintaining food sensory profiles while mitigating the risk of metabolic disorders [40]. The erythritol was incorporated into the formulation at a concentration of 3.24% (*w*/*w*), which aligns with clinical evidence demonstrating negligible gastrointestinal adverse effects [41]. Furthermore, this dosage fully complies with the World Health Organization (WHO) guidelines on non-sugar sweetener [42]. Therefore, the 3.24% erythritol concentration in bread formulation caused no risk to human health, while optimized parameters ensured both functional efficacy and consumer safety.

The contents of moisture, ash, protein, total fat, TDF, SDF, and IDF were measured, and carbohydrate content and caloric values were calculated; the results are shown in Table 2. The white bread had the highest moisture content of all breads, while bread 2CB had the lowest moisture content in all breads. With the addition of purple rice and chanterelle powder, the moisture content in all breads decreased. These results suggest that both purple rice and chanterelle powder could reduce the moisture content of bread, and especially chanterelle powder has greater influence than that of purple rice powder.

The ash content mainly contains mineral substances, which could increase nutritional richness in food and reduce the probability of hidden hunger occurrence [2]. The ash content of breads with purple rice and chanterelle powder was significantly higher than that of the white bread. With the proportion of purple rice and chanterelle powder increased, the ash content in bread increased.

The protein content in bread is not only an important nutritional evaluation indicator, but it also plays a crucial role in the fermentation, which contributes to form a fluffy tissue structure and improves the toughness and extensibility. The protein content of bread formulations varied significantly depending on the protein composition of substituted ingredients. Specifically, the partial substitution of wheat flour with chanterelle powder increased, whereas replacement with purple rice powder resulted in a net decrease in protein content compared to the control. Bread 2CB showed the highest protein content, and bread 10PB showed the lowest protein content.

The breads with purple rice powder had significantly lower fat content than that of white bread or bread containing only chanterelle powder. Compared to white bread, the total fat content of the breads with chanterelle powder slightly increased, while that of breads with purple rice powder decreased, and the fat content of breads both with purple rice and chanterelle powder was lower than that of white bread.

The content of TDF showed significant positive correlation with increasing wheat flour substitution levels. Bread 2C10PB showed the highest content at 4.98 g/100 g. Notably, the IDF content exhibited a progressive increase with higher substitution rates, while the SDF content had no significant differences among all formulations (*p* > 0.05). Both carbohydrate content and calories calculated displayed no significant differences (*p* < 0.05) compared with the control white bread.

The results are similar to those of Liu et al. [33] and Waewkum et al. [34] who reported the nutritional ingredient of chanterelle and purple rice. The protein and fat values in purple rice were slightly lower than those in wheat, while their contents in chanterelle mushroom were significantly higher than those in wheat. The content of fiber in chanterelle was significantly higher than that of purple rice, which was slightly higher than that of wheat flour.

### 3.2. Bread Quality Analysis

#### 3.2.1. Specific Volume

The specific volume of bread is a reflection of the degree of expansion and retention ability of the dough, which directly affects the appearance and taste of the bread [43]. The specific volume of breads is shown in Table 3. The results showed a decline in specific volume when purple rice and chanterelle powder were added. Compared with white bread, the specific volume of breads 1C10PB and 2C10PB was significantly decreased by 2.38 and 2.33 mL/g, respectively. The result of bread with purple rice powder was slightly lower than that of bread with chanterelle powder. This decrease may be attributed to dilution and interaction of the chanterelle and the purple rice powder with gluten, preventing the gluten network from forming as effectively as it does in white bread. A weaker gluten network reduces the gas holding capacity of bread, leading to a decrease in specific volume of the bread and causing the increased value of hardness and chewiness [44].

**Table 2:** Nutritional composition, HI, eGI, RDS, SDS, and RS content of the bread with different proportions of chanterelle and purple rice powder.

		White	1CB	2CB	5PB	10PB	1C5PB	2C5PB	1C10PB	2C10PB
Nutritional composition	Moisture (g/100 g)	36.74 ± 0.79 ^d^	35.29 ± 0.57 ^a,b^	34.82 ± 0.76 ^a^	36.54 ± 0.61 ^c,d^	36.23 ± 0.44 ^c,d^	36.22 ± 0.65 ^b,c^	35.93 ± 0.38 ^a,b^	35.54 ± 0.55 ^a,b^	35.22 ± 0.36 ^a,b^
	Ash (g/100 g)	2.69 ± 0.053 ^a^	2.74 ± 0.036 ^a,b^	2.77 ± 0.048 ^b^	2.81 ± 0.02 ^b,c^	2.92 ± 0.031 ^d^	2.87 ± 0.04 ^c,d^	2.90 ± 0.036 ^d^	3.01 ± 0.036 ^e^	3.05 ± 0.051 ^e^
	Protein (g/100 g)	14.67 ± 0.15 ^b,c^	15.60 ± 0.31 ^d^	15.95 ± 0.17 ^d^	14.61 ± 0.13 ^b^	14.23 ± 0.39 ^a^	14.83 ± 0.13 ^b,c^	15.04 ± 0.12 ^c^	14.81 ± 0.12 ^b,c^	15.02 ± 0.07 ^c^
	Total fat (g/100 g)	10.28 ± 0.25 ^c^	10.63 ± 0.24 ^d^	10.7 ± 30.19 ^d^	9.72 ± 0.20 ^b^	9.19 ± 0.21 ^a^	9.84 ± 0.04 ^b^	9.91 ± 0.32 ^b^	9.75 ± 0.09 ^b^	9.82 ± 0.08 ^b^
	TDF (g/100 g)	3.29 ± 0.19 ^a^	4.42 ± 0.11 ^b^	4.79 ± 0.06 ^d,e^	4.36 ± 0.21 ^b^	4.54 ± 0.06 ^b,c^	4.73 ± 0.05 ^c,d^	4.90 ± 0.07 ^d,e^	4.84 ± 0.08 ^d,e^	4.98 ± 0.06 ^e^
	SDF (g/100 g)	0.43 ± 0.05 ^a^	0.46 ± 0.05 ^a,b^	0.47 ± 0.04 ^a,b^	0.48 ± 0.03 ^a,b^	0.48 ± 0.05 ^a,b^	0.50 ± 0.04 ^a,b^	0.51 ± 0.03 ^a,b^	0.52 ± 0.03 ^b^	0.53 ± 0.04 ^b^
	IDF (g/100 g)	2.94 ± 0.09 ^a^	3.89 ± 0.08 ^b^	4.23 ± 0.09 ^c,d^	3.82 ± 0.07 ^b^	3.92 ± 0.04 ^b^	4.13 ± 0.13 ^c^	4.33 ± 0.11 ^d,e^	4.27 ± 0.14 ^d^	4.43 ± 0.06 ^e^
	Carbohydrates (%)	32.33 ± 0.81 ^b^	31.32 ± 1.01 ^a,b^	30.95 ± 0.70 ^a^	31.97 ± 0.77 ^a,b^	33.89 ± 0.71 ^c^	31.55 ± 0.74 ^a,b^	31.32 ± 0.48 ^a,b^	32.05 ± 0.58 ^a,b^	31.96 ± 0.34 ^a,b^
	Calories (kcal)	287.09 ± 2.81 ^a^	292.21 ± 2.20 ^b^	293.70 ± 3.47 ^b^	282.48 ± 3.60 ^a^	284.29 ± 2.56 ^a^	283.41 ± 2.86 ^a^	284.42 ± 1.42 ^a^	284.9 ± 2.09 ^a^	286.17 ± 0.84 ^a^
Hydrolysis index	HI(%)	95.50 ± 0.19 ^d^	90.15 ± 1.51 ^d^	89.03 ± 1.34 ^d^	81.19 ± 2.12 ^c^	74.48 ± 2.29 ^b^	89.66 ± 1.68 ^d^	88.66 ± 1.49 ^d^	56.82 ± 2.17 ^a^	57.33 ± 0.75 ^a^
	eGI (%)	97.71 ± 0.64 ^d^	86.76 ± 2.51 ^d^	84.93 ± 1.15 ^d^	78.19 ± 1.82 ^c^	72.38 ± 1.97 ^b^	86.35 ± 1.44 ^d^	84.62 ± 1.28 ^d^	57.17 ± 1.87 ^a^	57.61 ± 0.64 ^a^
Starch content	RDS (%)	48.69 ± 0.74 ^f^	46.27 ± 0.64 ^f^	43.74 ± 0.01 ^e^	39.03 ± 1.22 ^d^	35.23 ± 0.72 ^b^	37.17 ± 0.51 ^c^	35.93 ± 0.45 ^b,c^	32.86 ± 0.98 ^a^	32.03 ± 1.11 ^a^
	SDS (%)	29.11 ± 1.05 ^d^	28.78 ± 0.89 ^c,d^	27.85 ± 0.96 ^b,c^	26.96 ± 0.99 ^b,c^	25.73 ± 0.78 ^a,b^	27.38 ± 2.04 ^b,c^	27.91 ± 1.07 ^b,c^	24.98 ± 1.17 ^a,b^	23.74 ± 1.97 ^a^
	RS (%)	22.49 ± 2.37 ^a^	24.05 ± 0.66 ^a,b^	26.05 ± 0.43 ^b^	31.15 ± 0.74 ^c^	34.71 ± 0.65 ^d^	33.05 ± 0.16 ^c,d^	34.29 ± 0.23 ^d^	40.73 ± 0.74 ^e^	41.58 ± 2.31 ^e^

Note: TDF: total dietary fiber; SDF: soluble dietary fiber; IDF: insoluble dietary fiber; HI: hydrolysis index; eGI: expected glycemic index; RDS: rapidly digestible starch; SDS: slowly digestible starch; RS: resistant starch. 1CB: bread with 1% chanterelle mushroom powder; 2CB: bread with 2% chanterelle mushroom powder; 5PB: bread with 5% purple rice powder; 10PB: bread with 10% purple rice powder; 1C5PB: bread with 1% chanterelle mushroom and 5% purple rice powder; 2C5PB: bread with 2% chanterelle mushroom and 5% purple rice powder; 1C10PB: bread with 1% chanterelle mushroom and 10% purple rice powder; 2C10PB: bread with 2% chanterelle mushroom and 10% purple rice powder. Different letters (a, b, c, d, e, f) above the number show the significant differences between bread (*p* < 0.05), which a showed the lowest value.

#### 3.2.2. Color

The color of bread not only reflects the state of baking but also attracts and stimulates consumers to purchase bread with attractive colors [19]. The results of color value are presented in Table 3. The color intensity of bread crust was lower in formulations containing erythritol compared to conventional products, because erythritol cannot directly participate in Maillard reactions. There were significant differences (*p* < 0.05) in the color value of breads with different additions of purple rice and chanterelle powder compared to white bread. The lightness (L) values of bread crumb and crust in breads with chanterelle powder were similar to those of white bread, while the L value of breads with both purple rice and chanterelle powder and only purple rice powder significantly decreased (*p* < 0.05), which suggests that purple rice powder could decrease the L value of breads. The redness (a) value of the bread core increased with the addition of purple rice powder, with values between −3.58 and 5.21. In addition, the redness value of bread crust did not have a clear trend, but all the values were positive, with bread 10PB exhibiting the highest value.

The yellowness (b) value of bread crumbs increased with the addition of chanterelle powder and decreased with the addition of purple rice powder. The yellowness value of bread crust had no significant difference (*p* < 0.05). Bread 2C10PB had the highest color differential index (E), and bread 5PB showed the lowest value in bread core. The results indicate that the addition of purple rice and chanterelle powder made the bread become redder and more yellow. The color difference in breads may stem not only from inherent pigment among ingredients, but also from compositional disparities. While amino acids in wheat flour promote caramelization and browning, purple rice and chanterelle mushroom contain more soluble sugars and free amino acids, which causes more Maillard reactions during the baking process [44].

#### 3.2.3. Textural Characteristics of Breads

The breads exhibited significant differences (*p* < 0.05) in their textural characteristics when substituted with purple rice and chanterelle powder, as shown in Table 3. All breads with varying amounts of purple rice and chanterelle powder had higher hardness than that of white bread. Notably, bread 2C10PB had the highest hardness at 13.79 N. The research of Ortiz et al. indicated that there is a close relationship between bread hardness and specific volume; the decrease in the latter could lead to dough densification and increase in hardness [45]. Interestingly, bread 10PB exhibited lower hardness than that of 5PB. This may be attributed to synergistic interactions between branched amylopectin chains and gluten proteins, forming an entangled network structure. Furthermore, the higher contents of fiber and amylopectin in purple rice powder than those of wheat flour could improve crumb softness and moisture retention. These modifications resulted in enhanced textural properties in the high-substitution formulation [46].

Regarding springiness, the value of breads with purple rice and chanterelle powder had no significant difference (*p* < 0.05) from that of white bread, and the springiness of breads was slightly decreased. Furthermore, with the increase in the substitution of purple rice and chanterelle powder, the chewiness value significantly increased (*p* < 0.05), while the cohesiveness value slightly decreased (*p* < 0.05). The result is consistent with the research of Montes et al. (2024), which indicated that as the polyphenol content in bread increased, the hardness and chewiness value improved while the springiness and cohesiveness value diminished [47].

### 3.3. Total Phenolic Content and Total Flavonoid Content

**Figure 2:**
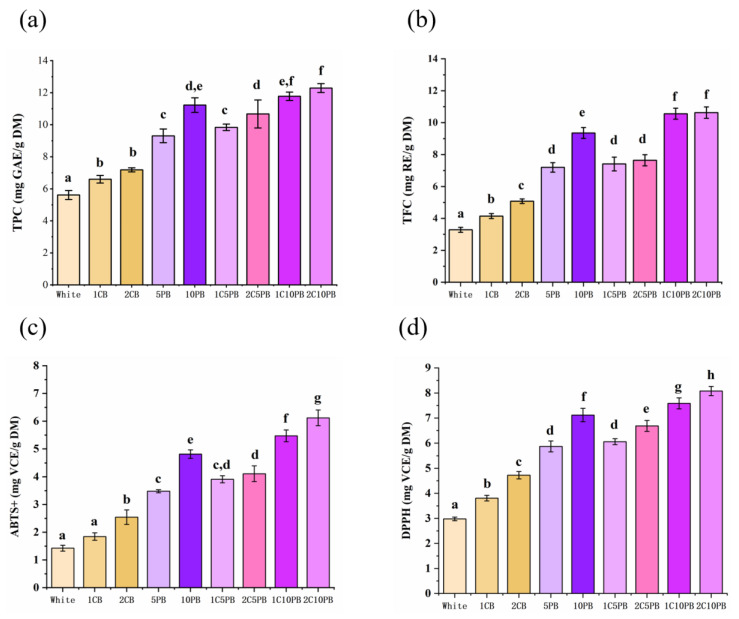
The total phenolic content (TPC) (**a**), the total flavonoid content (TFC) (**b**), ABTS (**c**), DPPH (**d**) of bread with different contents of chanterelle and purple rice powder. 1CB: bread with 1% chanterelle mushroom powder; 2CB: bread with 2% chanterelle mushroom powder; 5PB: bread with 5% purple rice powder; 10PB: bread with 10% purple rice powder; 1C5PB: bread with 1% chanterelle mushroom and 5% purple rice powder; 2C5PB: bread with 2% chanterelle mushroom and 5% purple rice powder; 1C10PB: bread with 1% chanterelle mushroom and 10% purple rice powder; 2C10PB: bread with 2% chanterelle mushroom and 10% purple rice powder. Different small letters above the column show the significant difference between breads (*p* < 0.05), where a shows the lowest value.

Total phenols and total flavonoids are not only important sources of antioxidants that could enhance nutritional richness, but they also affect the color, taste, and aroma of bread to increase sensory experience [48]. The total phenolic content (TPC) and total flavonoid content (TFC) is positively correlated with antioxidant capacity [36]. The results of the TPC and TFC determination are shown in Figure 2a,b. Replacing ingredients with both purple rice and chanterelle powder significantly increased the TPC and TFC values. Moreover, bread 2C10PB showed the highest values of TPC and TFC, which was more than twice that of white bread. According to the research of Ito et al. [10] and Liu et al. [33], chanterelle mushroom and purple rice both contain abundant phenolic and flavonoid compounds. Phenolic acids and anthocyanins are the main phenolic components in purple rice, while phenolic acids are the main phenolic components in chanterelle mushrooms. For flavonoid substances, naringin is the main component in chanterelle mushrooms, while cyanidin is the main component in purple rice [46,49].

### 3.4. Antioxidant Capacity Analysis

High antioxidant activity not only inhibits oil oxidation and microbial growth to extend the shelf life of bread but also helps protect the nutritional components like vitamins in bread, allowing them to be better absorbed and utilized by the human body [48]. The results of ABTS and DPPH values are shown in Figure 2c,d. Compared to the white bread, the bread with purple rice and chanterelle mushroom powder showed strong antioxidant activity. The trends of ABTS and DPPH activity were similar, and the value of DPPH was slightly higher than those of ABTS. As the proportion of purple rice and chanterelle mushroom powder increased, the values of ABTS and DPPH also increased. Bread 2C10PB showed the highest values of ABTS and DPPH, which suggests that adding purple rice and chanterelle mushroom can significantly increase the antioxidant capacity of bread. According to the studies by Ito et al. [10] and Tian et al. [16], purple rice is rich in antioxidant components like anthocyanin and polyphenols, which could eliminate free radicals in the body, reduce oxidative damage, and improve antioxidant capacity. Polysaccharide and polyphenol are rich in chanterelle mushrooms, which could directly enhance antioxidant capacity. Moreover, the Maillard reaction between amino acids in mushrooms and reducing sugars in bread, as well as antioxidant components like melanoidin, increase the antioxidant capacity [50].

### 3.5. In Vitro Starch Digestibility Analysis

**Figure 3:**
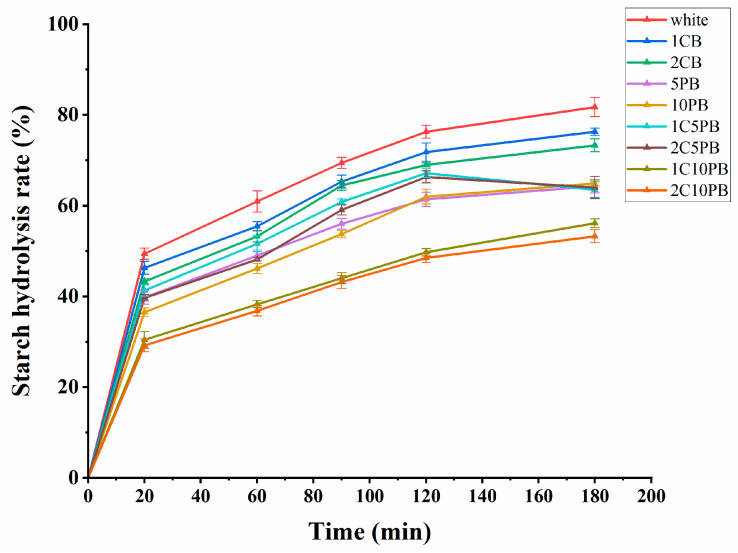
The starch hydrolysis rate of bread with different contents of chanterelle and purple rice powder. 1CB: bread with 1% chanterelle mushroom powder; 2CB: bread with 2% chanterelle mushroom powder; 5PB: bread with 5% purple rice powder; 10PB: bread with 10% purple rice powder; 1C5PB: bread with 1% chanterelle mushroom and 5% purple rice powder; 2C5PB: bread with 2% chanterelle mushroom and 5% purple rice powder; 1C10PB: bread with 1% chanterelle mushroom and 10% purple rice powder; 2C10PB: bread with 2% chanterelle mushroom and 10% purple rice powder.

The starch hydrolysis rate is an important indicator for evaluating the quality of bread. A low starch hydrolysis rate of bread not only slows down the aging rate of bread and better maintains its shape, but also controls blood sugar reactions and increases satiety [33]. The trend of the starch hydrolysis rate of breads is shown in Figure 3. It is shown that as digestion time increases, the starch hydrolysis rate of all breads gradually rises, but the rate of increase is slower for breads with purple rice and chanterelle powder. Bread with chanterelle powder slightly decreases starch hydrolysis rate over the whole time. This may be because chanterelle mushroom is high in dietary fiber which could inhibit the contact between amylase and starch to reduce the hydrolysis rate of starch, thereby reducing the glucose content in blood serum [51]. The starch hydrolysis rate of breads with purple rice powder is significantly lower than that of white bread over the whole time. This may be because polyphenol content rich in purple rice could bind to key digestive enzymes such as amylase and glucosidase, thus reducing their biological activity and slowing down the rate of glucose production [52]. The results show that with the proportion of wheat flour substitutes increased, the starch hydrolysis rate of bread decreases.

Resistant starch (RS) is defined as the starch that remains undigested in the human body for 2 h, and its physiological function is similar to dietary fiber. It cannot be digested in small intestine but can be fermented by intestinal microorganisms in the colon [53]. The fermentation products of RS can promote intestinal metabolism, improve intestinal barrier function, and help to prevent metabolic syndrome diseases such as type 2 diabetes mellitus and obesity [54]. The rapidly digestible starch (RDS) means starch broken down into glucose by the small intestine within 20 min, and slowly digestible starch (SDS) means starch digested within 20–120 min. There were significant differences in the RS content of breads (Table 2). With the addition of purple rice and chanterelle powder, the content of RDS and SDS decreased, and the content of RS significantly increased (*p* < 0.05). Compared to the white bread, the RS content in bread 2C10PB significantly increased (*p* < 0.05) from 22.49% to 41.58%. The increase may be due to the polyphenols both in purple rice and chanterelle, which could form complexes with amylose through hydrophobic interaction, thus reducing the starch resistance to digestion [55]. Notably, the content of RS in bread 5PB and 10PB also showed significant increase. This enhancement is attributed to the inherently elevated RS content in purple rice compared to wheat flour, the presence of dietary fibers that may impede starch gelatinization, and polyphenolic compounds that may chemically interact with inhibiting enzymatic digestion. These synergistic effects collectively contribute to improved RS content [56].

The hydrolysis index (HI) serves as a predictor of glycemic index (GI). GI is a measure of the rate at which carbohydrates release glucose into the bloodstream [8]. Research indicates that long-term consumption of high-GI foods increases the risk of type 2 diabetes [23], and the low GI diet is becoming more and more popular. The HI value of white bread was set to 100 and the values of HI and GI of breads were tested (Table 2). There was a significant reduction (*p* < 0.05) in HI and GI values of breads 1C10PB and 2C10PB. Moreover, with the proportion of purple rice and chanterelle powder increased, the HI value decreased, and bread 2C10PB showed the highest value of RS and the lowest value of GI. The RS content could slow down the rate of blood glucose rise, relatively stabilize insulin secretion, and reduce the value of GI [33].

### 3.6. Volatile Compound Analysis in Bread

The identification of flavor compounds in bread could optimize the formula and determine the preferred aroma components for consumers. The volatile compounds in breads substituted with purple rice and chanterelle powder are summarized in Table 4. Nine alcohols, eight aldehydes, seven eaters, one ketone, two acids, and nine hydrocarbons were detected in the breads with different proportions of purple rice and chanterelle powder. All the identified volatile compounds in bread contained more types of alcohols and aldehydes and fewer types of acids and ketones. Meanwhile, the relative content of volatile compounds had differences among different breads. Alcoholic compounds are an important component of bread aroma, which is mainly generated from oxidative degradation of lipids. Overall, nine alcoholic compounds were identified in breads, including ethyl alcohol, 3-methyl-1-butanol, and phenyl ethyl alcohol, which cause breads to have comprehensive aromas such as floral, fruity, and alcoholic. In particular, 1-octen-3-ol is a unique odorous compound found in bread with chanterelle powder, imparting the mushroom aroma. Among the eight aldehydes detected in the breads, nonanal, hexanal, and benzaldehyde are present in the highest content, which are mainly oxidation products from unsaturated fatty acids catalyzed by endogenous peroxidase and lipoxygenase [57]. All the breads have a comprehensive aromas profile that includes rose, fatty, and caramel, but the proportions of aldehydes are different in each bread, which leads to different aromas for each bread. Bread 1C10PB showed the highest content of nonanal, imparting aromas of rose and almond, while bread 2C10PB exhibited the highest aldehydes, resulting in the most pronounced aromas.

Esters are typically produced during the baking process of fermented dough and primarily impart a fruity aroma. Among the seven esters detected in breads, ethyl octanoate was the most abundant. Bread 10PB showed the highest esters content, resulting in the most pronounced fresh fruity and rose. In addition, decalactone imparted a peach aroma, which is unique to bread with purple rice powder, and bread 10PB showed the highest proportion of this compound. 2-nonone was the only ketone detected in the breads, contributing a sweet and fruit aroma, which was represented in the highest content in bread 1C10PB. Acid is mainly generated during the process of bread fermentations. Two acid compounds, 2-methylbutyric acid and 3-methylbutyric acid, were detected in bread, contributing to the overall cheesy and citrusy aroma. However, 3-methylbutyric acid, imparting a citrusy aroma, was not detected in bread with chanterelle powder.

Furthermore, nine hydrocarbons were identified, mainly containing saturated hydrocarbons, except for limonene. Saturated hydrocarbons generally do not impart significant odors, but limonene contributes to orange and lemon aromas; bread 1C10PB showed the highest proportion of limonene. Both 2-amylfuran and indole contribute floral and nutty aromas, respectively. The former was only detected in bread with chanterelle powder, and bread 2CB had the highest content of 2-amylfuran. Anisole, a unique fennel aroma, was detected in all breads, and was found in the highest content in bread 2C10PB.

In general, breads 10PB and 1C10PB showed the highest content of aroma, which showed higher value of sensory evaluation in taste. Moreover, bread with chanterelle mushroom powder showed a unique aroma of mushroom and nut, and bread with purple rice powder showed a special aroma with peach and citrus.

### 3.7. Sensory Attribute

**Figure 4:**
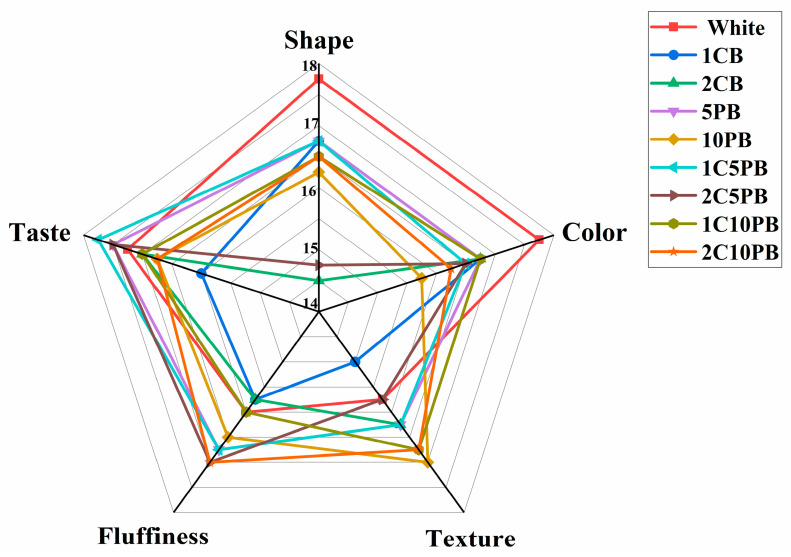
The sensory evaluation of bread with different contents of chanterelle and purple rice powder. 1CB: bread with 1% chanterelle mushroom powder; 2CB: bread with 2% chanterelle mushroom powder; 5PB: bread with 5% purple rice powder; 10PB: bread with 10% purple rice powder; 1C5PB: bread with 1% chanterelle mushroom and 5% purple rice powder; 2C5PB: bread with 2% chanterelle mushroom and 5% purple rice powder; 1C10PB: bread with 1% chanterelle mushroom and 10% purple rice powder; 2C10PB: bread with 2% chanterelle mushroom and 10% purple rice powder.

The shape, color, texture, fluffiness, and taste of the bread were evaluated by method of Adzqia et al. [38]. The sensory evaluation was structured into five parameters, each quantitatively evaluated ranging from 0 to 20 points (0 indicating extremely poor acceptance, 20 representing excellent performance) in accordance with the ISO 13299:2016 guidelines [39]. Since all sensory parameter scores fell within the range of 14–18, this interval was selected to enhance the visual clarity and data readability (Figure 4). White, 1CB, 5PB, and 1C5PB breads showed higher shape scores than others, exhibiting key attributes of superior smoothness of surface and appropriate volume in shape scoring. There was no significant difference (*p* > 0.05) in color acceptance score among all breads, with each displaying uniform luster in evaluation of sensory evaluators. Regarding texture, a slight difference was observed among the breads (*p* < 0.05), while bread 10PB obtained the highest score. In terms of fluffiness, all breads had fine and even evenly distributed pores, showing no significant difference (*p* > 0.05) in score. As for sensory evaluation, bread 1C5PB achieved higher consumer preference scores for aroma and flavor attributes, indicating superior olfactory and gustatory acceptability. A composite sensory score (CSS) was calculated to assess overall acceptability by five attributes (shape, color, texture, fluffiness, and taste). Both breads 10PB and 1C5PB achieved significantly higher CSS values, indicating superior overall sensory acceptability compared to other formulations.

### 3.8. Principal Component Analysis (PCA) and Correlation Analysis

**Figure 5:**
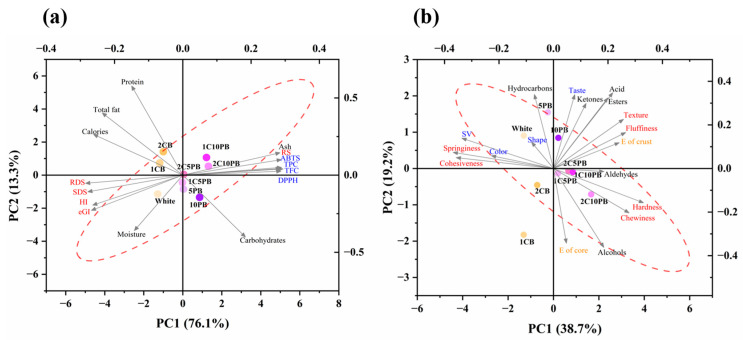
The score plot of principal components analysis (PCA) of bread with different contents of chanterelle and purple rice powder of different items (**a**,**b**). 1CB: bread with 1% chanterelle mushroom powder; 2CB: bread with 2% chanterelle mushroom powder; 5PB: bread with 5% purple rice powder; 10PB: bread with 10% purple rice powder; 1C5PB: bread with 1% chanterelle mushroom and 5% purple rice powder; 2C5PB: bread with 2% chanterelle mushroom and 5% purple rice powder; 1C10PB: bread with 1% chanterelle mushroom and 10% purple rice powder; 2C10PB: bread with 2% chanterelle mushroom and 10% purple rice powder. Moisture (%); Ash (%); Protein (%); Total fat (%); Carbohydrates (%); Calories (kcal); TPC, total phenolic content (mg GAE/g DM); TFC, total flavonoids content (mg RE/g DM); SV, specific volume (mL/g); HI, hydrolysis index (%); eGI, expected glycemic index (%); RDS, rapidly digestible starch (%); SDS, slowly digestible starch (%); RS, resistant starch (%); ∆E of core, color differential index of bread core; ∆E of crust, color differential index of bread crust; Hardness (N); Springiness (N); Cohesiveness (N); Chewiness (N).

The principal component analysis (PCA) of nutritional parameters, different types of starch content, antioxidant capacity, TPC, and TFC is shown in Figure 5a, which reveals that the percentage of variance explained by PC1 and PC2 was 76.1% and 13.3%, respectively. The ash content, resistant starch, ABTS, DPPH, TPC, and TFC are clustered at the top right corner of the image, suggesting positive correlations between PC1 and PC2. Another cluster at the lower left corner of the image contains the rapidly digestible starch, slowly digestible starch, hydrolysis index, and expected glycemic index, which exhibited negative correlation with PC1 and PC2. The protein, total fat, and calories had a long vector of PC1, showing negative correlation and significant contribution to PC1. Meanwhile, carbohydrates had a long vector of PC2, which showed negative correlation and significant contribution to PC2. Moreover, all samples showed less dispersion in the principal component direction, with high similarity in their values across the board.

The PCA of texture analysis value, color, specific volume, volatile compound analysis, and sensory evaluation value is shown in Figure 5b. The percentage of variance explained by PC1 and PC2 was 38.7% and 19.2%, respectively, with the contribution rate of 38.7% and 57.9%. The PCA incorporated 17 variables representing physicochemical parameters and volatile profiles. Moderate inter-variable correlations prevented any single latent factor from dominating the model. Consequently, PC1 and PC2 cumulatively accounted for less than 70% of the total variance; they still effectively discriminated key sample clusters. The specific volume, springiness, color, and cohesiveness showed a cluster at top left corner of the image, which indicated a negative correlation with PC1 and a positive correlation with PC2. In contrast, acid content in aroma, taste, and texture showed positive correlation with both PC1 and PC2, while alcohols, hardness, and chewiness showed positive correlation with PC1. In addition, breads 1C5PB, 2C5PB and 1C10PB had high cohesion, with the values showing high similarity among them. Conversely, bread 1CB showed large dispersion and significant difference in values compared to other samples.

**Figure 6:**
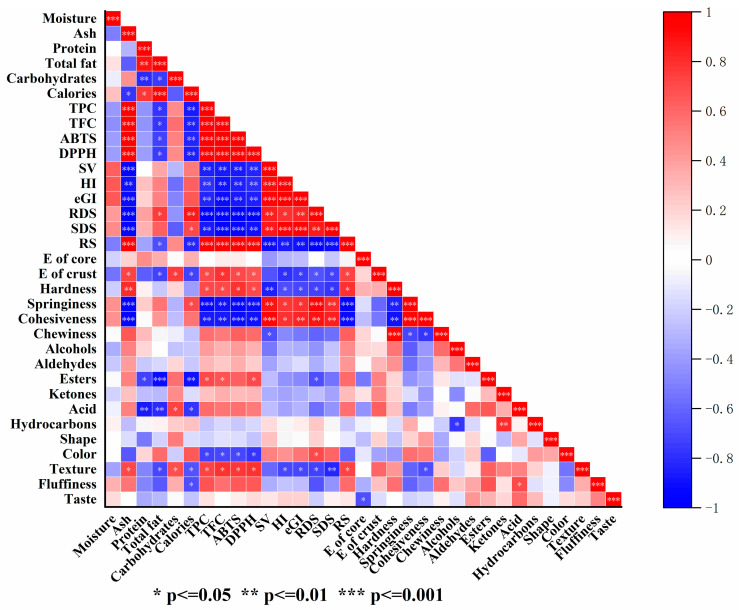
Pearson’s correlation of breads with different contents of chanterelle and purple rice powder. 1CB: bread with 1% chanterelle mushroom powder; 2CB: bread with 2% chanterelle mushroom powder; 5PB: bread with 5% purple rice powder; 10PB: bread with 10% purple rice powder; 1C5PB: bread with 1% chanterelle mushroom and 5% purple rice powder; 2C5PB: bread with 2% chanterelle mushroom and 5% purple rice powder; 1C10PB: bread with 1% chanterelle mushroom and 10% purple rice powder; 2C10PB: bread with 2% chanterelle mushroom and 10% purple rice powder. Moisture (%); Ash (%); Protein (%); Total fat (%); Carbohydrates (%); Calories (kcal); TPC, total phenolic content (mg GAE/g DM); TFC, total flavonoids content (mg RE/g DM); SV, specific volume (mL/g); HI, hydrolysis index (%); eGI, expected glycemic index (%); RDS, rapidly digestible starch (%); SDS, slowly digestible starch (%); RS, resistant starch (%); ∆E of core, color differential index of bread core; ∆E of crust, color differential index of bread crust; Hardness (N); Springiness (N); Cohesiveness (N); Chewiness (N).

The heat map illustrating the correlation of multiple variables is shown in Figure 6. In the map, the color is darker, and the correlation is stronger. The ash content had strong positive correlation with TPC, TFC, ABTS, DPPH, and RS. Conversely, it displayed strong negative correlations with SV, eGI, RDS, SDS, springiness, and cohesiveness. The total fat had strong positive correlations with calories, while the TPC, ABTS, and DPPH had strong negative correlations with RDS and springiness. The SV had a strong positive correlation with cohesiveness, but had a negative correlation with RS. Moreover, the RS had strong negative correlations with springiness and cohesiveness. The acid and ester contents in bread aroma had strong negative correlation with protein and total fat.

## 4. Conclusions

In this study, nine types of bread with different proportions of purple rice and chanterelle mushroom powder were manufactured, significantly enhancing the nutritional richness and functional characteristics of bread. Especially TPC, TFC, antioxidant capacity (ABTS and DPPH assays), and resistant starch content were significantly increased, while the hydrolysis index and expected glycemic index were significantly decreased through the addition of purple rice and chanterelle mushroom powder. Moreover, the texture characteristics and specific volume were changed with different proportions of purple rice and chanterelle mushroom powder. The hardness and chewiness increased, while the springiness and cohesiveness slightly decreased. The specific volume value decreased, which caused the scores of sensory evaluations to slightly lower compared to those of white bread, except for breads 5PB and 1C5PB. The aroma components were affected by the addition of purple rice and chanterelle mushroom powder, which increased the aroma content and contained unique flavors like nutty and fruity in the bread. Breads 5PB, 1C5PB, and 1C10PB had the highest sensory evaluation scores, and 1C10PB and 2CB showed the highest aroma components.

Breads 2C10PB, 1C10PB exhibited superior functional properties including antioxidant activity and glycemic index, while breads 1C10PB and 5PB demonstrated the practically optimal formulations when considering consumer-centric parameters such as sensory scores and specific volume. This finding reflects market requirements for balancing functional innovation with sensory acceptability. Consequently, based on physicochemical properties, functional performances, and consumer acceptance, bread 1C10PB formulation exhibited the highest comprehensive performance.

The bread with purple rice and chanterelle mushroom powder not only improved sensory attributes but also enhanced functional properties, which significantly improved nutritional quality and antioxidant capacity and reduced the hydrolysis index. Therefore, bread enriched with purple rice and chanterelle mushrooms is a great choice, which is a staple food not only because of its nutritional and functional characters, but also because of relieving the rate of hidden hunger. As natural food ingredients, purple rice and chanterelle mushrooms have exciting potential for improving the functional and nutritional profiles of bread.

The development of staple food formulations incorporating purple rice and chanterelle mushrooms provides a strategic approach to relieving the global health challenge of hidden hunger. Through synergistically enhancing nutritional density, boosting antioxidant capacity, and reducing the starch hydrolysis index, this approach successfully overcomes the limitations of conventional staple foods. Furthermore, this methodology demonstrates potential for other cereal-based staples, providing sustainable solutions for improving food fortification strategies. These advancements effectively bridge the gap between nutritional optimization and sensory acceptability.

## Figures and Tables

**Table 1:** Ingredients of the bread with different proportions of chanterelle and purple rice powder.

Ingredients	CCP (g)	PRP (g)	Wheat Flour (g)	Salt (g)	Erythritol (g)	Milk (g)	Yeast (g)	Butter (g)
White	0	0	250	2	15	175	5	15
1CB	2.5	0	247.5	2	15	175	5	15
2CB	5	0	245	2	15	175	5	15
5PB	0	12.5	237.5	2	15	175	5	15
10PB	0	25	225	2	15	175	5	15
1C5PB	2.5	12.5	235	2	15	175	5	15
2C5PB	5	12.5	232.5	2	15	175	5	15
1C10PB	2.5	25	222.5	2	15	175	5	15
2C10PB	5	25	220	2	15	175	5	15

Note: CCP: *Cantharellus yunnanensis Chiu* mushroom powder; PRP: purple rice powder. Salt: refined iodized table salt; milk: commercial Yili whole milk; butter: Anchor butter; wheat flour: commercially available Golden Dragon Fish high protein flour; yeast: commercial active dry yeast products (Lesaffre group, yeast viability of 10–20 billion CFU/g yeast). 1CB: bread with 1% chanterelle mushroom powder; 2CB: bread with 2% chanterelle mushroom powder; 5PB: bread with 5% purple rice powder; 10PB: bread with 10% purple rice powder; 1C5PB: bread with 1% chanterelle mushroom and 5% purple rice powder; 2C5PB: bread with 2% chanterelle mushroom and 5% purple rice powder; 1C10PB: bread with 1% chanterelle mushroom and 10% purple rice powder; 2C10PB: bread with 2% chanterelle mushroom and 10% purple rice powder.

**Table 3:** Textural, specific volume, and color of bread with different proportions of chanterelle and purple rice powder.

		White	1CB	2CB	5PB	10PB	1C5PB	2C5PB	1C10PB	2C10PB
Textural value	Hardness (N)	3.20 ± 0.54 ^a^	4.49 ± 0.68 ^b^	6.33 ± 0.36 ^d^	5.84 ± 1.79 ^c,d^	4.80 ± 0.78 ^b,c^	6.98 ± 0.55 ^d,e^	8.16 ± 0.69 ^e^	8.07 ± 0.90 ^e^	13.79 ± 1.40 ^f^
	Springiness (N)	0.91 ± 0.03 ^d^	0.89 ± 0.05 ^c,d^	0.87 ± 0.01 ^c,d^	0.86 ± 0.04 ^c^	0.82 ± 0.01 ^b^	0.80 ± 0.03 ^b^	0.79 ± 0.02 ^b^	0.75 ± 0.33 ^a^	0.73 ± 0.02 ^a^
	Cohesiveness (N)	0.66 ± 0.05 ^e^	0.66 ± 0.11 ^e^	0.55 ± 0.005 ^c,d^	0.59 ± 0.02 ^d^	0.56 ± 0.01 ^c,d^	0.56 ± 0.06 ^c,d^	0.52 ± 0.01 ^b,c^	0.48 ± 0.02 ^a,b^	0.44 ± 0.01 ^a^
	Chewiness (N)	1.88 ± 0.19 ^a^	2.65 ± 0.74 ^b,c^	3.02 ± 0.17 ^c,d^	2.95 ± 0.76 ^c,d^	2.19 ± 0.32 ^a,b^	3.19 ± 0.45 ^c,d^	3.35 ± 0.22 ^d^	2.89 ± 0.18 ^c,d^	4.29 ± 0.45 ^e^
Specific volume	SV (mL/g)	3.59 ± 0.16 ^d^	3.29 ± 0.93 ^c,d^	3.11 ± 0.07 ^b,c^	3.24 ± 0.01 ^c,d^	3.02 ± 0.09 ^b,c^	3.20 ± 0.08 ^b,c^	2.89 ± 0.24 ^b^	2.38 ± 0.21 ^a^	2.33 ± 0.26 ^a^
Color of bread core	L	98.22 ± 0.87 ^f^	96.27 ± 2.12 ^f^	96.33 ± 1.60 ^f^	83.38 ± 2.03 ^e^	69.08 ± 1.74 ^b^	79.66 ± 1.55 ^d^	76.58 ± 1.57 ^c^	69.91 ± 1.44 ^b^	65.71 ± 1.26 ^a^
	a	−3.58 ± 0.67 ^a^	−3.24 ± 0.31 ^a^	−2.92 ± 0.35 ^a^	0.18 ± 0.28 ^b^	4.47 ± 0.39 ^d,e^	1.92 ± 0.29 ^c^	3.12 ± 0.49 ^c,d^	4.29 ± 0.37 ^d,e^	5.21 ± 0.26 ^e^
	b	26.90 ± 1.12 ^e^	29.77 ± 0.80 ^f^	31.02 ± 1.22 ^f^	17.25 ± 0.63 ^b,c^	13.81 ± 0.25 ^a^	18.63 ± 0.34 ^c^	21.93 ± 3.68 ^d^	15.17 ± 0.17 ^a,b^	15.39 ± 0.34 ^a,b^
	E	24.34 ± 1.07 ^c^	26.85 ± 1.42 ^d^	27.84 ± 0.92 ^d,e^	15.86 ± 0.91 ^a^	24.95 ± 1.45 ^c,d^	19.11 ± 1.21 ^b^	23.72 ± 3.85 ^c^	24.76 ± 1.24 ^c,d^	29.72 ± 2.07 ^e^
Color of bread crust	L	86.39 ± 0.15 ^c^	87.32 ± 1.21 ^c^	68.07 ± 4.29 ^a^	74.57 ± 1.81 ^b^	64.46 ± 1.61 ^a^	75.54 ± 0.60 ^b^	74.53 ± 1.09 ^b^	64.44 ± 0.06 ^a^	64.54 ± 1.04 ^a^
	a	3.98 ± 0.42 ^b^	1.25 ± 0.19 ^a^	8.69 ± 1.55 ^e^	5.74 ± 1.24 ^b,c^	8.15 ± 1.49 ^d,e^	4.56 ± 0.27 ^b,c^	3.58 ± 1.67 ^a,b^	8.67 ± 0.32 ^e^	6.51 ± 0.71 ^c,d^
	b	33.98 ± 1.49 ^d^	31.71 ± 1.30 ^c,d^	16.76 ± 4.04 ^a^	28.04 ± 1.74 ^b,c^	27.43 ± 1.63 ^b,c^	25.49 ± 0.91 ^b^	30.75 ± 1.46 ^c,d^	29.03 ± 0.29 ^b,c^	25.74 ± 2.62 ^b^
	E	30.98 ± 1.42 ^b,c^	28.31 ± 1.47 ^a,b^	25.26 ± 3.53 ^a^	30.19 ± 2.67 ^b^	36.87 ± 2.57 ^d^	27.36 ± 0.43 ^a,b^	32.08 ± 2.02 ^b,c^	38.05 ± 0.23 ^d^	35.42 ± 0.99 ^c,d^

Note: SV: Specific volume. L: lightness; a: redness; b: yellowness; E: color differential index. Different letters (a, b, c, d, e, f) above the number showed the significant differences between bread (*p* < 0.05), which a showed the lowest value. 1CB: bread with 1% chanterelle mushroom powder; 2CB: bread with 2% chanterelle mushroom powder; 5PB: bread with 5% purple rice powder; 10PB: bread with 10% purple rice powder; 1C5PB: bread with 1% chanterelle mushroom and 5% purple rice powder; 2C5PB: bread with 2% chanterelle mushroom and 5% purple rice powder; 1C10PB: bread with 1% chanterelle mushroom and 10% purple rice powder; 2C10PB: bread with 2% chanterelle mushroom and 10% purple rice powder.

**Table 4:** Relative abundance (%) of volatile compounds of the bread with different proportions of chanterelle and purple rice powder.

Chemical Types	Volatile Compounds	Odor	White	1CB	2CB	5PB	10PB	1C5PB	2C5PB	1C10PB	2C10PB
Alcohols	Ethyl alcohol	Alcohol, spicy	8.17 ± 0.32	12.76 ± 0.48	12.23 ± 1.45	13.06 ± 0.19	11.35 ± 0.41	3.34 ± 0.69	8.12 ± 1.15	13.26 ± 0.87	12.32 ± 0.74
	3-Methyl-1-Butanol	Malty, alcohol	7.29 ± 0.88	3.51 ± 0.88	10.73 ± 1.68	9.53 ± 0.31	10.13 ± 0.28	9.92 ± 0.42	10.41 ± 0.51	11.96 ± 0.12	13.26 ± 0.86
	Phenylethyl alcohol	Rose, honey	3.13 ± 0.56	5.47 ± 0.45	6.31 ± 0.33	3.23 ± 0.83	2.59 ± 0.37	4.70 ± 0.16	5.43 ± 0.42	3.92 ± 0.29	3.10 ± 0.45
	Hexanol	Green, flower, sweet	0.79 ± 0.16	0.49 ± 0.12	0.56 ± 0.10	0.69 ± 0.06	0.93 ± 0.05	0.51 ± 0.10	0.49 ± 0.04	1.06 ± 0.11	1.09 ± 0.14
	2,3-Butanediol	Neutral, soft	0.72 ± 0.42	nd	nd	0.29 ± 0.09	0.54 ± 0.06	0.12 ± 0.04	0.58 ± 0.09	0.70 ± 0.06	0.86 ± 0.04
	1-Heptanol	Grassy, vegetable	nd	9.91 ± 0.21	nd	0.64 ± 0.16	0.40 ± 0.18	0.41 ± 0.09	0.81 ± 0.09	0.35 ± 0.11	0.37 ± 0.13
	3,7-Dimethyl-1-Octanol	NF	0.39 ± 0.06	0.20 ± 0.07	0.31 ± 0.09	nd	nd	nd	nd	nd	nd
	3-None-1-ol	Flower, fruity	0.33 ± 0.06	0.53 ± 0.14	0.94 ± 0.27	0.28 ± 0.09	0.43 ± 0.05	0.51 ± 0.09	0.55 ± 0.08	0.64 ± 0.13	0.68 ± 0.15
	1-Octen-3-ol	Mushroom	nd	0.16 ± 0.04	0.24 ± 0.04	nd	nd	0.11 ± 0.07	0.19 ± 0.04	0.16 ± 0.05	0.21 ± 0.06
Aldehydes	Nonanal	Rose, almondy	1.64 ± 0.15	1.24 ± 0.12	1.66 ± 0.18	1.35 ± 0.12	1.55 ± 0.13	1.19 ± 0.19	1.04 ± 0.14	1.59 ± 0.06	1.47 ± 0.23
	Hexanal	Balsamic, grassy	1.47 ± 0.14	1.63 ± 0.17	1.16 ± 0.11	0.40 ± 0.19	0.55 ± 0.14	0.85 ± 0.19	1.14 ± 0.24	0.67 ± 0.15	1.54 ± 0.27
	Benzaldehyde	Almondy, caramel	1.13 ± 0.11	1.37 ± 0.21	2.45 ± 0.28	0.65 ± 0.17	0.79 ± 0.14	0.92 ± 0.16	1.33 ± 0.13	1.46 ± 0.09	1.64 ± 0.13
	Decanal	Sweet flower	0.52 ± 0.14	0.69 ± 0.20	0.54 ± 0.14	0.41 ± 0.07	0.49 ± 0.07	0.36 ± 0.04	0.31 ± 0.04	0.41 ± 0.09	0.47 ± 0.08
	2-Nonenal	Grassy, citrusy	0.51 ± 0.16	0.45 ± 0.15	0.53 ± 0.16	0.46 ± 0.09	0.59 ± 0.19	0.44 ± 0.12	0.54 ± 0.11	0.47 ± 0.07	0.50 ± 0.12
	2-Octenal	Nut, bake	0.41 ± 0.16	0.39 ± 0.24	0.52 ± 0.16	nd	nd	0.25 ± 0.08	0.36 ± 0.04	0.22 ± 0.06	0.25 ± 0.04
	Phenylacetaldehyde	Rose	0.18±0.04	0.37 ± 0.07	0.81 ± 0.14	0.18 ± 0.04	0.21 ± 0.04	0.56 ± 0.09	0.62 ± 0.07	0.45 ± 0.10	0.61 ± 0.11
	2-Heptenaldehyde	Citrusy	0.14 ± 0.05	nd	nd	0.17 ± 0.07	0.24 ± 0.05	0.26 ± 0.07	0.36 ± 0.06	0.46 ± 0.08	0.31 ± 0.07
Esters	Ethyl octanoate	Fruity	2.42 ± 0.11	2.30 ± 0.23	3.02 ± 0.27	2.36 ± 0.23	3.03 ± 0.25	1.89 ± 0.19	2.20 ± 0.22	2.70 ± 0.21	1.97 ± 0.22
	Ethyl decanoate	Fruity, brandy	0.35 ± 0.05	0.49 ± 0.05	0.67 ± 0.06	0.50 ± 0.07	0.79 ± 0.10	0.49 ± 0.07	0.49 ± 0.11	0.63 ± 0.04	0.53 ± 0.06
	Ethyl lactate	fruity	0.15 ± 0.02	nd	nd	nd	nd	nd	nd	nd	nd
	Octyl formate	NF	nd	0.22 ± 0.06	nd	0.24 ± 0.04	0.61 ± 0.03	nd	nd	0.71 ± 0.03	nd
	Propyl nonyl lactone	Almondy, peach	nd	nd	0.17 ± 0.04	0.20 ± 0.04	0.27 ± 0.04	0.20 ± 0.04	0.18 ± 0.04	nd	0.20 ± 0.05
	Phenylethyl acetate	Rose, honey	0.19 ± 0.04	0.37 ± 0.07	0.77 ± 0.08	0.18 ± 0.04	0.22 ± 0.05	0.57 ± 0.09	0.60 ± 0.11	0.46 ± 0.12	0.60 ± 0.09
	Decalactone	Fruity, peach	nd	nd	nd	0.22 ± 0.04	0.30 ± 0.08	0.21 ± 0.06	0.23 ± 0.05	0.25 ± 0.05	0.27 ± 0.06
Ketones	2-Nonanone	fruity	0.42 ± 0.07	nd	0.44 ± 0.12	0.43 ± 0.13	0.46 ± 0.07	nd	0.33 ± 0.07	0.56 ± 0.09	0.40 ± 0.08
Acid	2-Methylbutyric acid	Cheese	0.35 ± 0.04	0.54 ± 0.11	0.19 ± 0.04	0.50 ± 0.09	0.30 ± 0.07	0.40 ± 0.10	0.16 ± 0.07	0.33 ± 0.06	0.39 ± 0.09
	3-Methylbutyric acid	Citrusy	0.29 ± 0.054	nd	nd	0.22 ± 0.08	0.82 ± 0.19	0.29 ± 0.11	0.17 ± 0.05	0.37 ± 0.12	0.33 ± 0.12
Hydrocarbons	Dodecane	Insipid	3.39 ± 0.49	4.64 ± 0.69	6.13 ± 0.56	4.49 ± 0.48	5.56 ± 0.24	3.95 ± 0.40	3.73 ± 0.55	4.77 ± 0.48	4.42 ± 0.45
	Undecane	Insipid	2.59 ± 0.21	2.95 ± 0.26	3.53 ± 0.28	3.00 ± 0.18	3.52 ± 0.17	2.97 ± 0.21	2.78 ± 0.48	3.71 ± 0.45	2.62 ± 0.32
	Decane	Insipid	2.33 ± 0.44	1.70 ± 0.72	1.97 ± 0.63	1.74 ± 0.57	1.95 ± 0.51	1.67 ± 0.48	1.38 ± 0.51	1.90 ± 0.32	nd
	2,6-Dimethyloctane	Insipid	0.55 ± 0.11	nd	0.18 ± 0.04	0.20 ± 0.07	0.43 ± 0.06	nd	nd	0.27 ± 0.07	0.34 ± 0.06
	Tetradecane	Insipid	0.29 ± 0.04	0.45 ± 0.06	0.62 ± 0.04	0.44 ± 0.12	0.67 ± 0.09	0.33 ± 0.13	0.52 ± 0.17	0.51 ± 0.16	0.54 ± 0.12
	2,6,6-Trimethyl Octane	Insipid	0.25 ± 0.04	nd	0.34 ± 0.09	0.29 ± 0.04	nd	nd	0.30 ± 0.08	nd	0.36 ± 0.14
	4-Methyldodecane	Insipid	0.29 ± 0.12	0.26 ± 0.09	0.33 ± 0.11	0.17 ± 0.05	0.32 ± 0.04	0.25 ± 0.08	0.32 ± 0.13	0.39 ± 0.10	0.45 ± 0.07
	2-Methyltetradecane	Insipid	0.22 ± 0.04	0.28 ± 0.08	0.47 ± 0.09	0.32 ± 0.14	0.42 ± 0.09	0.28 ± 0.06	0.33 ± 0.11	0.36 ± 0.08	0.35 ± 0.10
	Limonene	Flower, fruity, orange	0.51 ± 0.03	0.95 ± 0.11	1.01 ± 0.13	0.59 ± 0.06	1.33 ± 0.14	0.74 ± 0.13	0.84 ± 0.14	1.34 ± 0.11	0.65 ± 0.11
Others	2-Amylfuran	Flower, nut	0.55 ± 0.11	0.43 ± 0.13	0.56 ± 0.09	nd	nd	0.11 ± 0.03	0.35 ± 0.07	0.20 ± 0.06	0.50 ± 0.11
	Indole	Flower, nut	nd	0.28 ± 0.05	0.41 ± 0.07	nd	nd	nd	nd	nd	nd
	Anisole	Fennel	0.11 ± 0.02	0.18 ± 0.04	0.26 ± 0.08	0.25 ± 0.07	0.30 ± 0.10	0.19 ± 0.05	0.27 ± 0.07	0.22 ± 0.08	0.29 ± 0.04

Note: nd: not detected; NF: not found. 1CB: bread with 1% chanterelle mushroom powder; 2CB: bread with 2% chanterelle mushroom powder; 5PB: bread with 5% purple rice powder; 10PB: bread with 10% purple rice powder; 1C5PB: bread with 1% chanterelle mushroom and 5% purple rice powder; 2C5PB: bread with 2% chanterelle mushroom and 5% purple rice powder; 1C10PB: bread with 1% chanterelle mushroom and 10% purple rice powder; 2C10PB: bread with 2% chanterelle mushroom and 10% purple rice powder.

## Data Availability

The original contributions presented in this study are included in the article. Further inquiries can be directed to the corresponding authors.
